# Crizotinib-Induced Abnormal Signal Processing in the Retina

**DOI:** 10.1371/journal.pone.0135521

**Published:** 2015-08-13

**Authors:** Toshiyuki Ishii, Shunichiro Iwasawa, Ryota Kurimoto, Akemi Maeda, Yuichi Takiguchi, Makoto Kaneda

**Affiliations:** 1 Department of Physiology, Nippon Medical School, 1-1-5 Sendagi, Bunkyo-ku, Tokyo 113–8602, Japan; 2 Department of Medical Oncology, Graduate School of Medicine, Chiba University1-8-1, Inohana Chuo-ku, Chiba 260–8670, Japan; 3 Tokyo Medical Care and Welfare Vocational School, 1-11-11 Hatchobori, Chuo-ku, Tokyo, 104–0032, Japan; Dalhousie University, CANADA

## Abstract

Molecular target therapy for cancer is characterized by unique adverse effects that are not usually observed with cytotoxic chemotherapy. For example, the anaplastic lymphoma kinase (ALK)-tyrosine kinase inhibitor crizotinib causes characteristic visual disturbances, whereas such effects are rare when another ALK-tyrosine kinase inhibitor, alectinib, is used. To elucidate the mechanism responsible for these visual disturbances, the responses to light exhibited by retinal ganglion cells treated with these agents were evaluated using a C57BL6 mouse *ex vivo* model. Both crizotinib and alectinib changed the firing rate of ON and OFF type retinal ganglion cells. However, the ratio of alectinib-affected cells (15.7%) was significantly lower than that of crizotinib-affected cells (38.6%). Furthermore, these drugs changed the response properties to light stimuli of retinal ganglion cells in some of the affected cells, i.e., OFF cells responded to both ON and OFF stimuli, etc. Finally, the expressions of *ALK* (a target receptor of both crizotinib and alectinib) and of *MET* and *ROS1* (additional target receptors of crizotinib) were observed at the mRNA level in the retina. Our findings suggest that these drugs might target retinal ganglion cells and that the potency of the drug actions on the light responses of retinal ganglion cells might be responsible for the difference in the frequencies of visual disturbances observed between patients treated with crizotinib and those treated with alectinib. The present experimental system might be useful for screening new molecular target agents prior to their use in clinical trials.

## Introduction

The development of drugs for targeted therapy has revolutionized cancer therapy. Some of these drugs promise significant responses for patients with cancers expressing the molecular target. However, these drugs have specific and unique adverse effects that differ from those of cytotoxic agents. For example, skin disorders including rashes, pruritus, and acne are seen in patients treated with gefitinib [1], erlotinib [2] and cetuximab [3], while severe diarrhea is seen in patients treated with afatinib [4].

The standard therapy for patients with non-small-cell lung cancer (NSCLC) without specific target molecules is platinum doublets, resulting in a median survival time of almost 10–14 months and a response rate of 20% to 35% [5,6]. In contrast, NSCLC expressing specific target molecules is highly sensitive to molecular target drugs. In patients with NSCLC with mutated epidermal growth factor receptor (*EGFR*), EGFR-tyrosine kinase inhibitors, including gefitinib and erlotinib, have enabled a significantly longer survival time of about 30 months and higher response rates of 70% to 80% [7,8].

Anaplastic lymphoma kinase (ALK) is a target molecule for the treatment of several cancers, including NSCLC, inflammatory myofibroblastic tumor, and anaplastic large-cell lymphoma [9,10]. The fusion of the *ALK* gene with others, including echinoderm microtubule-associated protein-like 4 (*EML4*) and kinesin family member 5B (*KIF5B*), is observed in about 5% of patients with NSCLC [11,12]. In NSCLC with *ALK* rearrangements, the fused gene can lead to tumorigenesis in the absence of other oncogenes, such as mutated *EGFR* and *KRAS* [13]. These *ALK* rearrangements are an important target for treatment.

Crizotinib is the first established tyrosine kinase inhibitor targeting ALK, the mesenchymal-epithelial transition (MET), and repressor of silencing 1 (ROS1) [9,14,15]. In large-scale, randomized clinical trials for patients with NSCLC with *ALK* rearrangements, about 60% of the patients achieved an objective response [16,17,18]. Furthermore, crizotinib is also effective against NSCLC with *ROS1* rearrangements [19]. However, a specific profile of adverse effects is seen in patients treated with crizotinib, with nausea, liver damage, and neutropenia being commonly seen. Above all, unique visual disorders can occur. Mild visual disturbances have been reported in some patients (41%-62%) participating in clinical trials [16,17,18]. Patients with these disorders usually complain of trails of light following objects that are in motion relative to the observer, particularly during changes in ambient lighting from dark to light [16]. These events generally start within two weeks of the administration of crizotinib and improve with time without the need to discontinue treatment. However, these adverse effects can affect the quality of life of patients.

The cause of these visual disturbances, which are unique to crizotinib, remains unknown. In recent studies examining patients treated with alectinib, which is a newly established and more specific inhibitor of ALK, the frequency of adverse effects (including visual disorders) was significantly lower than that for crizotinib [20,21]. In addition, visual disorders did not occur in patients treated with an inhibitor of MET, which is one of the targets of crizotinib [22].

Since the visual pathway is a complex system, visual disorders can occur at multiple levels, such as the retina, the lateral geniculate nucleus, and the primary visual cortex. In the present study, to examine whether the retina can be a target of drugs and whether the actions of drugs on retinal ganglion cells can predict differences in the incidences of adverse effects, we compared the actions of crizotinib and alectinib on the activities of retinal ganglion cells in the mouse retina using multi-electrode array recording.

## Materials and Methods

### Preparations

The research protocol was approved by the Animal Experiments Ethical Review Committee of Nippon Medical School. The samples and methods used in the present experiments were essentially the same as those used in a previous paper [23]. Mice (C57BL6, 6 weeks to 5 months old, male or female) dark adapted for 30–60 min were killed by cervical dislocation. Retina was isolated under dim red light and kept in oxygenated Ringer’s solution (in mM: NaCl, 120; KCl, 3.1; CaCl_2_, 2; MgSO_4_, 1; NaH_2_PO_4_, 0.5; NaHCO_3_, 23; glucose, 20; pH adjusted to 7.4–7.5 when bubbled with 95% O_2_−5% CO_2_) in the dark at room temperature until use. During the recordings, the retina was continuously superfused with oxygenated Ringer’s solution.

### Current recordings

A piece of retina (3–6 mm per side) was placed ganglion cell side-down on a multi-electrode array (MED-P2H07A; Panasonic) and perfused with oxygenated Ringer’s solution (1 mL/min). The recorded extracellular action potentials were amplified 1000-fold, band-pass filtered (0.1–10 kHz), and sampled with a computer at 20 kHz. The action potentials of individual cells were identified using an off-line analysis with custom-developed software [24]. Drugs were applied by bath application. Crizotinib was purchased from LC Laboratories (MA, USA). Alectinib was obtained from Chugai Pharmaceutical (Tokyo, Japan). The concentrations of crizotinib (1.0 μM) and alectinib (1.0 μM) used in the present study were almost equal to the Cmax (maximum plasma concentration) values, which were measured in repeated-dose studies [21,25]. All the experiments were performed at room temperature.

### Classification of retinal ganglion cells

The retina was stimulated using spatially uniform white illumination switching between 0.7 mW/ m^2^ (dark stimulation) and 17 mW⁄ m^2^ (bright stimulation) at 0.5 Hz. The action potentials recorded for 60 s (30 cycles of light stimulation) before drug application were pooled and used to calculate the mean firing rates during bright stimulation (*f*_*bright*_) and dark stimulation (*f*_*dark*_). When the mean firing rate was less than 1 Hz before drug application, the cells were not used for further analysis. For each stimulation, the action potentials in a 1-second window of bright stimulation or dark stimulation, respectively, starting at 50 ms after onset were used to calculate the *f*_*bright*_ or *f*_*dark*_ values_._ The stimulus preference index (*SPI*) was calculated using the following formula:

$$SPI=\frac{pref}{pref+n.pref}$$ (1) ,

where *pref* is the mean firing rate with the higher frequency between that for *f*_*bright*_ and that for *f*_*dark*_, and *n*.*pref* is the mean firing rate with the lower frequency. If the *SPI* was equal to or higher than 0.67 and *pref* was the rate for *f*_*bright*_, the cells were classified as ON type retinal ganglion cells (ON-cells). If the *SPI* was equal to or higher than 0.67 and *pref* was the rate for *f*_*dark*_, the cells were classified as OFF type retinal ganglion cells (OFF-cells). Cells not classified as either ON- or OFF-cells were classified as ON-OFF type retinal ganglion cells (ON-OFF cells). In some of the experiments, we also calculated the *SPI* during and after the application of drugs using a method similar to that described above.

### Data analysis

To assess the actions of the drugs on the firing rate, we calculated the mean firing rate using the following method. First, we calculated the mean firing rate (*r*_*pre*_) and the standard deviation (*s*_*pre*_) before drug application using the same data set as that used for the *SPI* analysis. To calculate *s*_*pre*_, we sectioned the data before drug application into 10 bins (3 cycles each [6 s]) and used the following equation:

$$s_{pre}=\sqrt{\frac{1}{n-1}\sum_{i=1}^{n}\left(x_{i}-r_{pre}\right)}$$ (2) ,

where *n* is total number of bins and *x* is the mean firing rate of the individual bin. We also calculated the mean firing rate during (*r*_*drug*_) and after (*r*_*post*_) drug application. The index, X, was calculated using the following formula:

$$X=\left|r_{drug}-\frac{r_{pre}+r_{post}}{2}\right|$$ (3) .

If *X* was larger than twice the value of *s_pre,_* (corresponding to *P* < 0.05), the action of the drug on the firing rate was judged as “significant”.

To assess the stimulus information transferred by the retinal ganglion cells, the spike triggered average stimulus (STA or *F*(*T*)) was calculated using the light stimulus information. In this calculation, light stimulation for 1 s preceding a spike was binned with a resolution of 50 ms. The stimulus during a time interval (*T*) before the onset of an individual action potential was averaged according to a previously reported protocol (Dayan and Abbott, 2001) and the following equation:

$$F(T)=\frac{1}{n}\sum_{i=1}^{n}s(t_{i}-T)$$ (4) ,

where n is the number of spikes, *t* is the time when the spikes occurred, and *s*(*t*) is the light intensity as a function of time. The light intensity for this analysis was defined as 1 for bright stimulation and 0 for dark stimulation.

### Statistical analysis

When the groups had equal variance, we adopted a parametric test. For samples with different variances, we adopted a nonparametric test. Comparisons of variance between groups were performed using the F test.

### Reverse transcription PCR and quantitative RT-PCR

Total RNA was extracted from mouse retina (C57BL6, 8 weeks) using ISOGEN reagent (Nippongene, Japan). The RNA was pooled in a PCR tube containing a ribonuclease (RNase) inhibitor (TaKaRa) and Primer Mix (ReverTra Ace qPCR RT Kit; Toyobo). The PCR tube was heated at 75°C for 5 min and cooled on ice for 1 min; RT Enzyme Mix and 5xRT Buffer (ReverTra Ace qPCR RT Kit; Toyobo) were then added. Reverse transcription (RT) into cDNA was performed using TaKaRa PCR Thermal Cycler Dice Standard (TaKaRa). RT was performed at 30°C for 10 min and then at 42°C for 60 min. After stopping the reaction by heating at 75°C for 15 min, the reaction mixture was kept at 4°C and stored at -80°C until use. A relative quantitative PCR (qPCR) analysis was performed using the SYBR Premix Ex Taq II (TaKaRa) and Thermal Cycler Dice real-time System Single (TaKaRa) following the manufacturer’s instructions. *ß*-actin (*ACTB*), a housekeeping gene, was used as an internal reference, and distilled water was used as a negative control. The primer sequences, product sizes, and accession numbers are listed in Table 1. For each cDNA, relative qPCR was performed in triplets. The relative expression levels of mouse anaplastic lymphoma kinase (*ALK*), *MET*, and *ROS1* were compared with that of *ACTB* using the ΔΔCT method. The PCR products were separated using electrophoresis on 2% agarose gels and were visualized using ethidium bromide staining under UV irradiation.

**Table 1:** Oligonucleotide primers used for qPCR.

Gene		Primer sequence (5’–3’)	Size (bp)	Accession number
*ALK*	Forward	ctgtggctgtcagtacctagag	135	NM_007439.2
	Reverse	ctgtagatatctcgggccatcc		
*MET*	Forward	ggatggctgtggagagaaag	75	NM_008591.2
	Reverse	ggagccttcattgtgaggag		
*ROS1*	Forward	cggaccaaaagagtcagtcc	77	NM_011282.2
	Reverse	cctgtcttagaggagtctcagg		
*ACTB*	Forward	ctaaggccaaccgtgaaaag	104	NM_007393.3
	Reverse	accagaggcatacagggaca		

## Results

### Actions of crizotinib and alectinib in the retina

**Fig 1:**
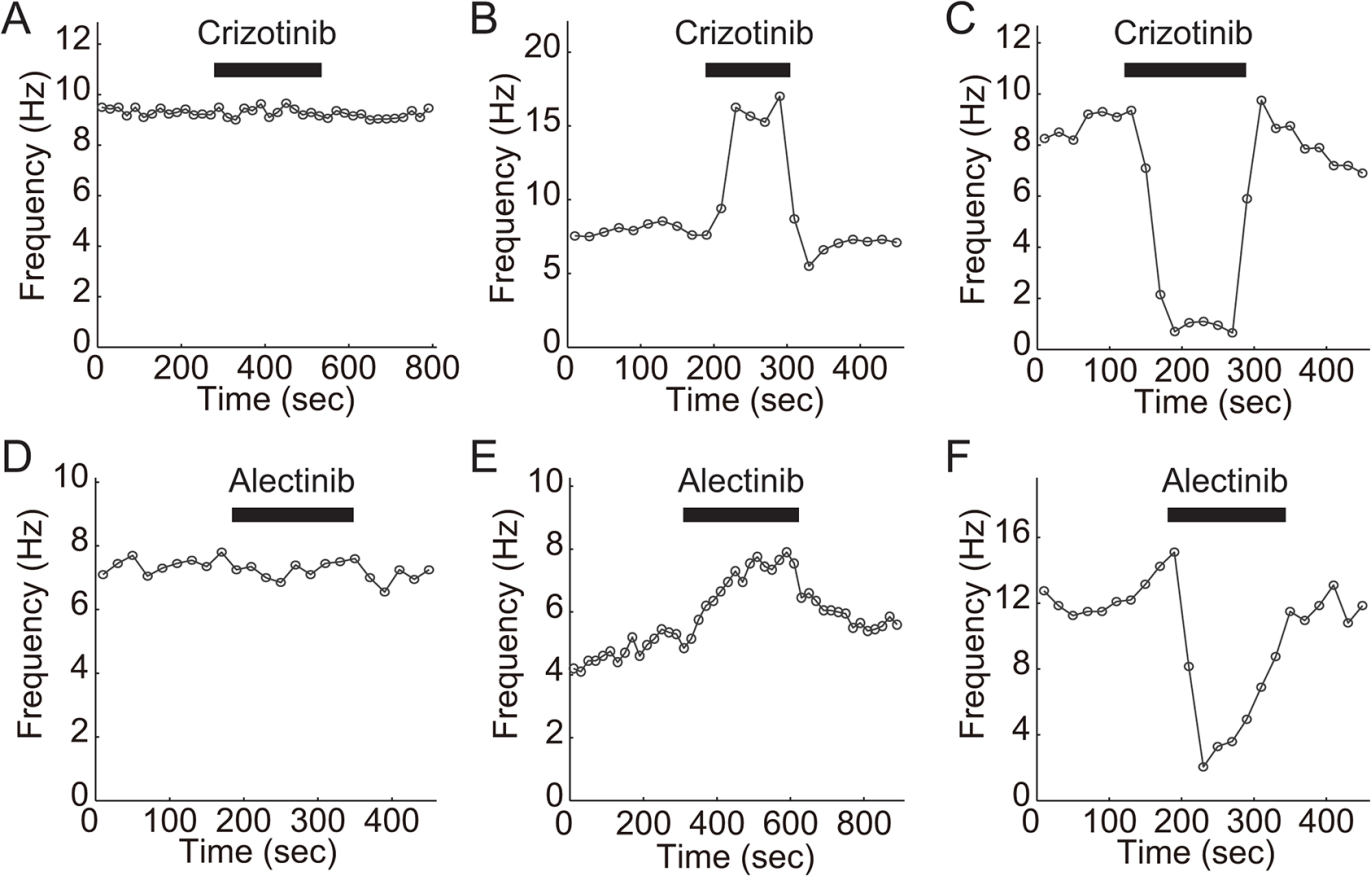
Effect of crizotinib (A-C) or alectinib (D-F) on firing rate of retinal ganglion cells. The timing of the drug application is indicated by the bar above the traces. The drug concentration was 1.0μM for both crizotinib and alectinib. The results for no change-type (A, D), increase-type (B, E), and decrease-type (C, F) cells are shown. The retina was repeatedly exposed to a set of light stimuli (1-s bright stimulation and 1-s dark stimulation at a frequency of 0.5 Hz). The ordinate shows the average firing rate for 10 cycles of stimuli (20 s).

To examine whether crizotinib modifies visual function at the retinal level, we collected 101 retinal ganglion cells (48 ON-cells, 47 OFF-cells, and 6 ON-OFF cells) (Table 2) and examined the actions of crizotinib on the firing rate. Among the 101 cells that were examined, crizotinib changed the firing rate in 44 cells (43.6%). The actions of crizotinib on the firing rate were classified into no change type (Fig 1A), increase type (Fig 1B), and decrease type (Fig 1C). Among the cells that showed a change in the firing rate (increase or decrease type), the change in the firing rate occurred soon after the application of crizotinib. The actions of crizotinib on the firing rate were reversible.

**Table 2:** Effects of crizotinib or alectinib on firing rate.

Firing rate	ON-cell		OFF-cell		ON-OFF cell	
	Crizotinib n (%)	Alectinib n (%)	Crizotinib n (%)	Alectinib n (%)	Crizotinib n (%)	Alectinib n (%)
Increase	5 (10.4)	0 (0.0)	10 (21.3)	13 (27.7)	0 (0.0)	0 (0.0)
Decrease	14 (29.2)	6 (16.2)	12 (25.5)	2 (4.3)	3 (50.0)	0 (0.0)
No change	29 (60.4)	31 (83.8)	25 (53.2)	32 (68.1)	3 (50.0)	3 (100.0)
Total	48 (100.0)	37 (100.0)	47 (100.0)	47 (100.0)	6 (100.0)	3 (100.0)

We performed a similar analysis for alectinib to compare the severity of visual disorders induced by crizotinib and alectinib. Among the 87 cells that were examined (37 ON-cells, 47 OFF-cells, and 3 ON-OFF cells) (Table 2), alectinib changed the firing rate (increase or decrease type) in 21 cells (24.1%). The actions of alectinib on the firing rate were also classified into three types (Fig 1D–1F). The actions of alectinib on the firing rate occurred soon after the application of alectinib and were reversible.

Since both crizotinib and alectinib showed three different types of actions on the firing rate, we directly compared whether the actions of crizotinib and the actions of alectinib on the firing rate were the same in individual cells. We selected cells in which we were able to examine the actions of both crizotinib and alectinib successfully in the same cell and classified these cells into 5 categories (Table 3). Among the 70 cells (30 ON-cells, 37 OFF-cells, and 3 ON-OFF cells) that were examined, the actions of the drugs on the firing rate were the same in 52 cells (Category “S” + “N”), while opposite actions on the firing rate were observed in only 2 cells (Category “O”). None of the cells showed a change in the firing rate in response to alectinib only (Category “A”). The remaining 16 cells showed a change in the firing rate in response to crizotinib only (Category “C”). These findings suggest that the actions of crizotinib and alectinib on the firing rates of individual cells are basically the same (correlation coefficient, 0.51; *P* < 0.001). However, a significant difference in the number of affected cells was observed between these two drugs (*P* < 0.01, Chi-squared test). Alectinib affected 11 cells (15.7%, Category “S” + “A”), while crizotinib affected 27 cells (38.6%, Category “S” + “C”). The difference in the number of affected cells between the crizotinib and alectinib groups may explain the lower incidence of visual disorders induced by alectinib.

**Table 3:** Comparison of actions of crizotinib or alectinib on firing rate.

Firing rate						
Crizotinib	Alectinib	ON-cell (n)	OFF-cell (n)	ON-OFF cell (n)	Total (n)	Category
Increase	Increase	0	5	0	5	S
Decrease	Decrease	3	1	0	4	S
Increase	Decrease	0	1	0	1	O
Decrease	Increase	0	1	0	1	O
Increase	No change	3	4	0	7	C
Decrease	No change	5	4	0	9	C
No change	Increase	0	0	0	0	A
No change	Decrease	0	0	0	0	A
No change	No change	19	21	3	43	N
Total		30	37	3	70	

Category S: both crizotinib and alectinib had the same effect on the firing rate. Category O: the drugs had opposite effects on the firing rate. Category C: only the effect of crizotinib was significant. Category A: only the effect of alectinib was significant. Category N: neither crizotinib nor alectinib had an effect.

### Actions of crizotinib and alectinib on stimulus preference

**Fig 2:**
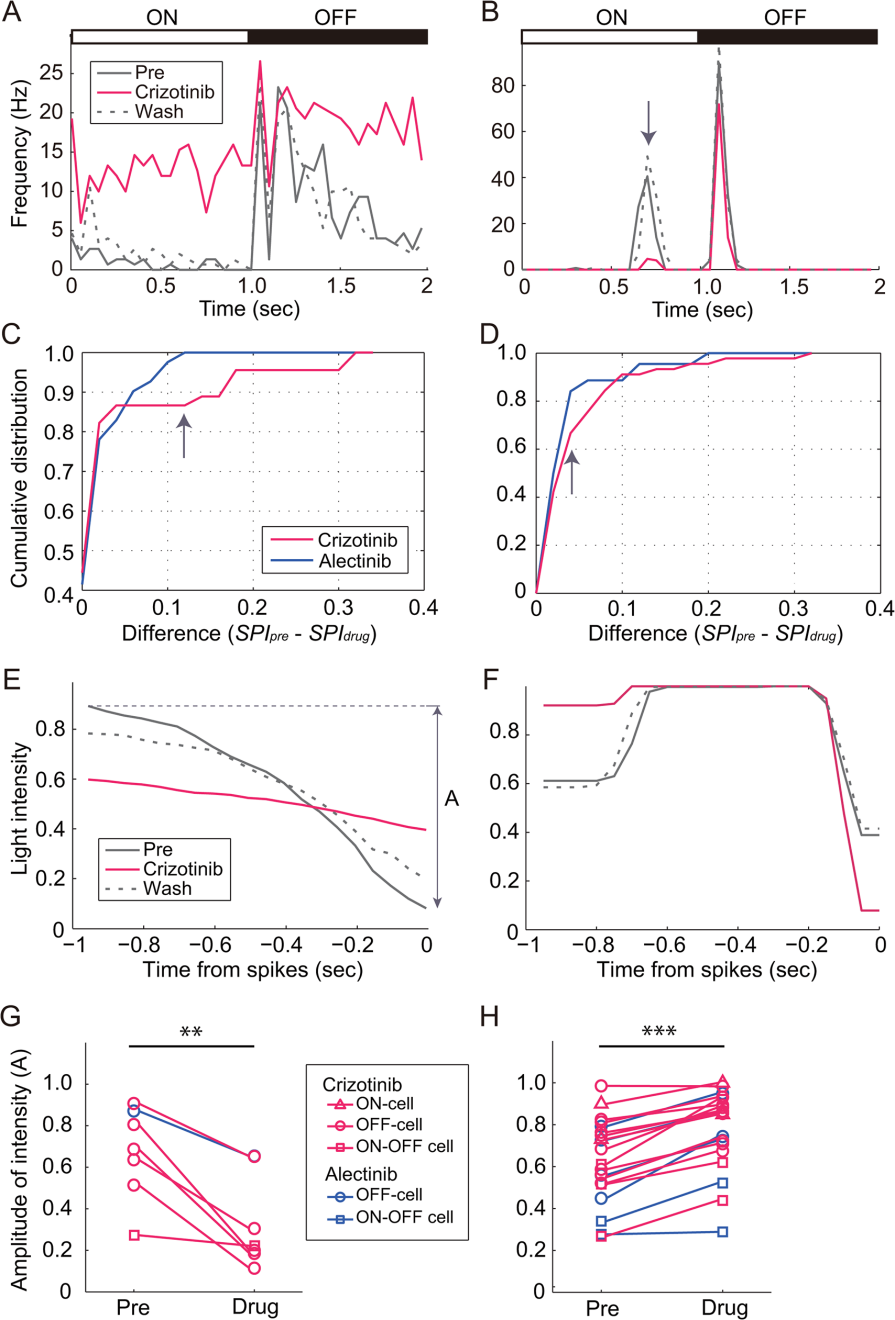
Effect of crizotinib and alectinib on stimulus preference of retinal ganglion cells. (A and B) Post-stimulus time histogram before (pre), during (crizotinib), and after (wash) the application of 1.0μM of crizotinib in an OFF-cell (A) and in an ON-OFF cell (B). The bin size for the histogram was 50 ms. (C and D) Cumulative distributions of the differences in *SPI* for crizotinib and alectinib. The difference in *SPI* was calculated from the *SPI* values evaluated before and during drug application. The cells were divided into “Decrease” type (C) and “Increase” type (D) according to the change in the *SPI*. The difference in (D) was an absolute value. (E and F) STA before (pre), during (crizotinib), and after (wash) the application of crizotinib in the cells shown in Figs 2A (E) and B (F). The amplitude “A” was defined as the difference in light intensity between the maximum and the minimum (double-headed arrow). (G and H) Plot of the amplitude “A” before (pre) and during drug application (drug) for the cells shown in Table 4. The cells were divided into “Decrease” type (G) and “Increase” type (H). **P < 0.01; *** *P* < 0.001; paired *t*-test.

Next, we examined the actions of crizotinib and alectinib on stimulus preference. The effects of drugs on stimulus preference might be related to the severe visual disorder, since a change in the preference of OFF-cells implies that dark stimuli are not being detected properly (and *vice versa* for ON-cells and bright stimuli). Fig 2A and 2B shows the averaged firing rates of representative cells exhibiting severe *SPI* changes induced by crizotinib. In Fig 2A, the cell responded to dark stimuli before drug application (black solid line) (*SPI* = 0.92). However, once crizotinib was applied to the cell, the cell began to respond to both dark and bright stimuli (red line) (*SPI* = 0.61), indicating that the OFF-cell had begun to behave like an ON-OFF cell. The actions of crizotinib on the firing rate were reversible (black dotted line) (*SPI* = 0.82). In contrast, the cell shown in Fig 2B originally responded to both dark and bright stimuli (black solid line) (*SPI* = 0.61). During the application of crizotinib, the cell did not respond to bright stimuli (red line) (*SPI* = 0.91), suggesting that the cell type had changed from an ON-OFF cell to an OFF-cell. The actions of crizotinib on the firing rate were also reversible (black dotted line) (*SPI* = 0.59). Thus, the change in *SPI* was bidirectional.

To assess the effect of crizotinib or alectinib on the *SPI*, we calculated the *SPI* before (*SPI*_*pre*_) and during (*SPI*_*drug*_) drug application for all the cells shown in Table 2. First, we classified the cells into three groups based on the difference between *SPI*_*pre*_ and *SPI*_*drug*_ for individual cells. “Decrease” means that the drug decreased the *SPI* (46 cells for crizotinib and 41 cells for alectinib), while “Increase” means that the drug increased the *SPI* (44 cells for crizotinib and 44 cells for alectinib). In some of the cells, the difference between *SPI*_*pre*_ and *SPI*_*drug*_ was not significant (“No change” type; 11 cells for crizotinib and 2 cells for alectinib). For both the “Decrease” and “Increase” types, the cumulative distributions of crizotinib and alectinib overlapped each other at some point. However, at the point where the cumulative distribution of alectinib had almost reached a plateau (arrow), that of crizotinib was not yet saturated. The difference in the cumulative distributions of crizotinib and alectinib means that crizotinib induced a greater change in the *SPI* than alectinib. Table 4 shows the numbers of cells exhibiting a larger difference in the *SPI* than the difference indicated by the arrow (Fig 2C and 2D). Here, we extracted severely affected cells from the “Decrease” and “Increase” categories, and cells with a smaller difference than that indicated by the arrow were classified as a “Mild change” group. Twenty (2 + 0 + 0 + 0 + 9 + 5 + 3 + 1, in Table 4) cells were severely affected by crizotinib, while only 7 (0 + 0 + 4 + 1 + 2 + 0) cells were severely affected by alectinib. The number of severely affected cells differed significantly between the drugs (*P* < 0.05, Chi-squared test), supporting the higher incidence of severe visual disorder in patients treated with crizotinib. A large change in the *SPI* was not always associated with a change in the firing rate (8 out of 20 cells for crizotinib, 4 out of 7 cells for alectinib).

**Table 4:** Effects of crizotinib or alectinib on *SPI*.

*SPI*	ON-cell		OFF-cell		ON-OFF cell	
	Crizotinib n (%)	Alectinib n (%)	Crizotinib n (%)	Alectinib n (%)	Crizotinib n (%)	Alectinib n (%)
Increase	2 (4.2)	0 (0.0)	9 (19.1)	4 (8.5)	3 (50.0)	2 (66.7)
Decrease	0 (0.0)	0 (0.0)	5 (10.6)	1 (2.1)	1 (16.7)	0 (0.0)
Mild or No change	46 (95.8)	37 (100.0)	33 (70.2)	42 (89.4)	2 (33.3)	1 (33.3)
Total	48 (100.0)	37 (100.0)	47 (100.0)	47 (100.0)	6 (100.0)	3 (100.0)

### Further analysis of cells with large SPI differences

How does the change in the *SPI* modify the transferred visual information? To visualize the transferred visual information in the presence and absence of drugs, we used the STA. The cell (“Decrease” type) shown in Fig 2A tended to respond when the light intensity decreased greatly (Fig 2E), indicating that the cell mainly fired during dark stimulation before drug application (pre). During the application of crizotinib, the cell tended to respond to relatively steady light stimuli, indicating that the cell fired in response to both dark and bright stimuli. To analyze the actions of the drugs on the transferred information, the amplitude “A” (Fig 2E, double-headed arrow) was defined as the difference between the maximum and minimum STA intensity. The amplitude decreased in all the cells and became significantly small during drug application (n = 7, *P* < 0.01, paired *t*-test, Fig 2G). On the other hand, the cell (“Increase” type) shown in Fig 2B initially had a low *SPI*, indicating that the cell responded to both dark and bright stimuli (Fig 2F). During the application of crizotinib, the cell lost its response to bright stimuli (Fig 2B, arrow); consequently, an increase in the *SPI* was observed. In the “Increase” cell type, the amplitude “A” became significantly large during drug application (n = 20, *P* < 0.001, paired *t*-test, Fig 2H). These changes indicate that the property of the transferred visual information differed during drug application. Since only a few cells were examined for alectinib, we did not compare the actions between crizotinib and alectinib.

Ganglion cells must correctly detect light stimuli (signals) under the presence of various background light sources (noise). Here, we regarded the firing rate for the preferred stimulation as the signal and that for non-preferred stimulation as noise and calculated the signal to noise ratio (S/N) of the cells, as shown in Table 4. After drug application, the S/N ratio decreased significantly from 16.13 ± 8.23 to 2.63 ± 0.77 (mean ± SEM) in 6 OFF-cells, which exhibited a reduced *SPI* (*P* < 0.05, Wilcoxon signed-rank test), while it increased significantly from 5.71 ± 0.84 to 18.26 ± 5.55 (mean ± SEM) in 13 OFF-cells, which exhibited an increased *SPI* (*P* < 0.001, Wilcoxon signed-rank test). Therefore, crizotinib and alectinib might affect the spontaneous noise level.

### Expression of mRNAs targeted by crizotinib or alectinib

**Fig 3:**
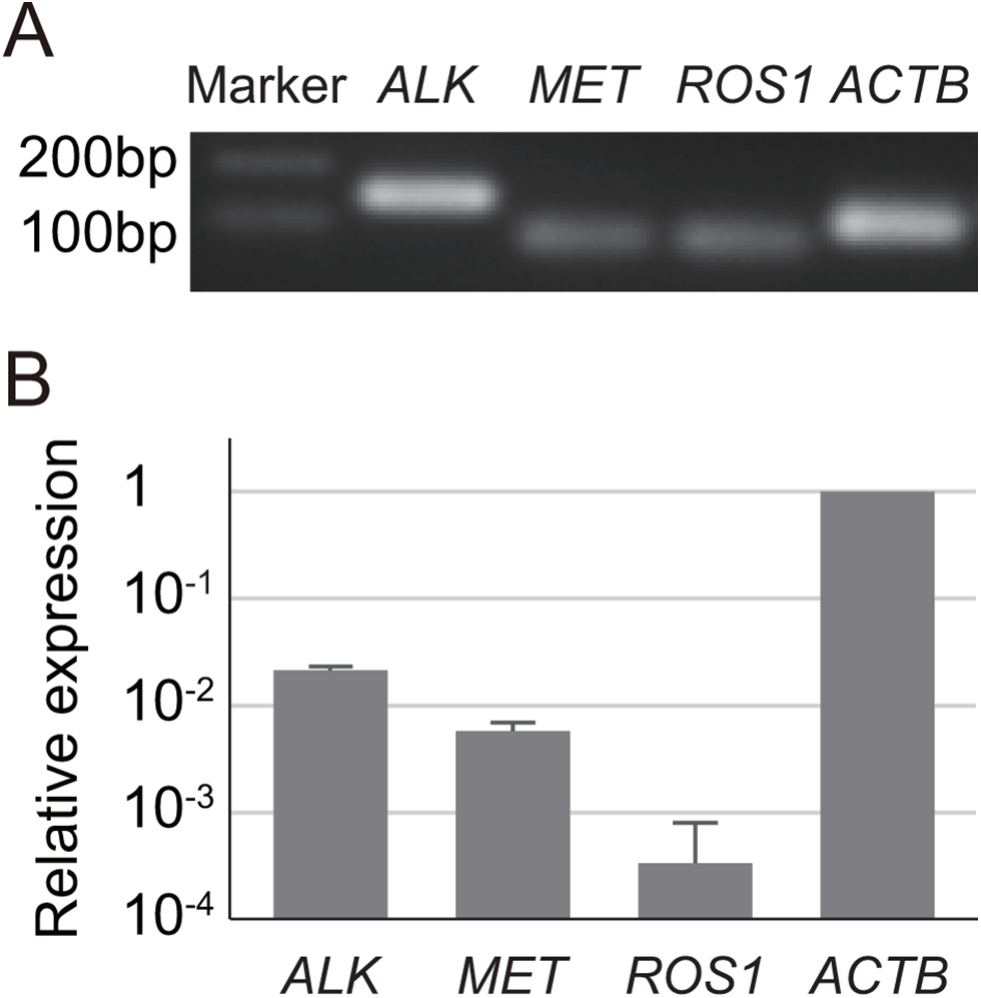
Expressions of *ALK*, c*MET*, and *ROS1* mRNA in adult mouse retina. (A) Specificity of qPCR products as detected using electrophoresis. (B) Relative mRNA expression levels. The *ALK*, c*MET*, and *ROS1* signals were normalized according to the *ACTB* signal. Error bars, s.d.; number of animals = 3.

To examine whether target receptors for crizotinib or alectinib exist in mouse retina, we performed qPCR for *ALK*, a target receptor of both crizotinib and alectinib, and for *MET* and *ROS1*, two additional target receptors of crizotinib. For *ALK*, *MET*, and *ROS1*, single bands of the expected sizes were detected (Fig 3A, Table 1). Among the three target receptors, the expression level of *ALK* was the highest and that of *ROS1* was the lowest. The expression levels of *ALK*, *MET*, and *ROS1* were lower than that of *ACTB* (Fig 3B). The absence of additional signal amplification was confirmed by the dissociation curve (data not shown).

## Discussion

In this experiment, we examined whether crizotinib or alectinib could induce visual disorders at the level of the retinal ganglion cells. We found that both crizotinib and alectinib disturbed the signal processing of retinal ganglion cells and that the functional disturbance induced by crizotinib was stronger than that induced by alectinib. Our data strongly support the idea that functional disturbance of the retinal ganglion cells is one possible source of visual disorder and that the magnitude of the functional disturbance may be a useful indicator for predicting the severity of visual disorders induced by newly synthesized drugs.

### Effects of crizotinib and alectinib on firing rate and stimulus preference

The application of crizotinib or alectinib reversibly changed the firing rate and the stimulus preference to light stimulation in some cells. Changes in the firing rate were significantly more common during treatment with crizotinib than during treatment with alectinib. In addition, significantly more cells exhibited drastic changes in stimulus preference during treatment with crizotinib than during treatment with alectinib.

Clinically, visual disorders induced by crizotinib are more severe than those induced by alectinib. Experimentally, the firing rate is generally considered to convey information regarding the stimulus intensity [26,27]. For example, the firing rate of ON-cells increases with increasing light intensity. Thus, the firing rate reflects a quantitative change in the light stimuli. On the other hand, the stimulus preference reflects a qualitative change in light stimuli for the following reason. We used the *SPI* to characterize the ganglion cells based on the visual information that they transferred. When ON-cells exhibited a large reduction in *SPI*, the cells were re-classified as ON-OFF cells. The change in the *SPI* meant that the ON-cells could not respond to the proper light stimuli at the proper timing. Therefore, a change in stimulus preference can be interpreted as a qualitative change in light stimuli. We concluded that a qualitative index was a better indicator for assessing the severity of side effects, although large changes were observed both quantitatively and qualitatively during treatment with crizotinib.

### Molecular targets of crizotinib and alectinib

Why was there a difference in the severity of visual disorders between crizotinib and alectinib? We confirmed the expressions of *ALK*, *MET*, and *ROS1* mRNA (receptor tyrosine kinases that serve as therapeutic targets in non-small-cell lung cancers [19,28]), in the mouse retina (Fig 3). Immunoreactivity for ALK protein has been observed in mice [29]. Both crizotinib and alectinib inhibit ALK at nanomolar concentrations, but the incidence of visual disorder is higher for crizotinib. Therefore, the high frequency of visual disorder induced by crizotinib is unlikely to be attributable to ALK inhibition. This idea is consistent with previous findings indicating that an ALK-selective inhibitor did not produce significant changes on an electroretinogram [29]. Crizotinib also inhibits MET and ROS1 at nanomolar concentrations [25], while the inhibitory actions of alectinib on MET and ROS1 only occur at micromolar concentrations or higher [30,31]. However, the presence of MET [29] and ROS1 has not been confirmed at the protein level in the mouse retina. Therefore, it will be interesting to see if the actions of crizotinib on MET or ROS1 in the retina play a key role in the severe visual disorder.

### Possible visual disorder in the retina

How do *SPI* disturbances modify our signal processing of visual scenes? We observed drastic changes in the *SPI* in a few OFF-cells. Since OFF-cells respond to dark stimuli and ON-cells respond to bright stimuli, edges with a sharp contrast are detected by the coordination of both ON- and OFF-cells. When the *SPI* of OFF-cells is drastically reduced, edges with a sharp contrast cannot be detected clearly, leading to confusion in higher visual centers. Therefore, *SPI* disturbances in the retina may be a source of the symptoms (such as diplopia and blurred vision) that sometimes occur in patients treated with crizotinib, and the transfer of incorrect signals from the retina may create confusion in higher visual centers.

The present study has some limitations that should be considered. In addition to using a mouse model, we did not examine the effects of the agents on higher visual centers, such as the lateral geniculate nucleus or the primary visual cortex. Thus, the present results may not concisely explain the visual disturbances experienced by patients treated with crizotinib. Furthermore, although the concentrations of the agents used in the present study were comparable to the Cmax level used in clinical situations, many unidentified factors may affect the actual drug concentrations in the retina. These factors may include differences in the concentration and/or clearance between vascular and intraocular regions as well as the protein binding capacity of the agents. All these issues should be taken into consideration when translating the results of our *ex vivo* experiments into clinical situations.

In conclusion, ALK-tyrosine kinase inhibitors affected the signal processing of light at the retinal ganglion cell level in an *ex vivo* mouse model, and the effects of crizotinib were severer than those of alectinib, possibly explaining the difference in adverse effects (visual disturbances) observed in clinical situations.
